# Genetic reduction of the extracellular matrix protein versican attenuates inflammatory cell infiltration and improves contractile function in dystrophic *mdx* diaphragm muscles

**DOI:** 10.1038/s41598-020-67464-x

**Published:** 2020-07-06

**Authors:** Natasha L. McRae, Alex B. Addinsall, Kirsten F. Howlett, Bryony McNeill, Daniel R. McCulloch, Nicole Stupka

**Affiliations:** 1 Centre for Molecular and Medical Research, School of Medicine, Deakin University, Waurn Ponds, VIC 3216 Australia; 2 Department of Physiology and Pharmacology, Karolinska Institute, Stockholm, Sweden; 3 Institute of Physical Activity and Nutrition, School of Exercise and Nutrition Sciences, Deakin University, Geelong, VIC 3216 Australia; 4 Present Address: Australian Institute for Musculoskeletal Science (AIMSS), University of Melbourne and Western Health, St Albans, VIC Australia; 5 Present Address: Department of Medicine -Western Health, Melbourne Medical School, University of Melbourne, St Albans, VIC Australia

**Keywords:** Experimental models of disease, Physiology

## Abstract

There is a persistent, aberrant accumulation of V0/V1 versican in skeletal muscles from patients with Duchenne muscular dystrophy and in diaphragm muscles from *mdx* mice. Versican is a provisional matrix protein implicated in fibrosis and inflammation in various disease states, yet its role in the pathogenesis of muscular dystrophy is not known. Here, female *mdx* and male hdf mice (haploinsufficient for the versican allele) were bred. In the resulting F1 *mdx*-hdf male pups, V0/V1 versican expression in diaphragm muscles was decreased by 50% compared to *mdx* littermates at 20–26 weeks of age. In *mdx*-hdf mice, spontaneous physical activity increased by 17% and there was a concomitant decrease in total energy expenditure and whole-body glucose oxidation. Versican reduction improved the ex vivo strength and endurance of diaphragm muscle strips. These changes in diaphragm contractile properties in *mdx*-hdf mice were associated with decreased monocyte and macrophage infiltration and a reduction in the proportion of fibres expressing the slow type I myosin heavy chain isoform. Given the high metabolic cost of inflammation in dystrophy, an attenuated inflammatory response may contribute to the effects of versican reduction on whole-body metabolism. Altogether, versican reduction ameliorates the dystrophic pathology of *mdx*-hdf mice as evidenced by improved diaphragm contractile function and increased physical activity.

## Introduction

Duchenne Muscular Dystrophy (DMD) is an X-linked, paediatric disease arising from a mutation in the dystrophin (*DMD*) gene leading to the loss of expression of dystrophin and the dystrophin associated protein complex (DAPC)^1^, which renders muscles highly vulnerable to degeneration. Increasing fibrosis and excessive inflammation compromise muscle repair leading to muscle wasting and expansion of the extracellular matrix^2,3^. Skeletal, respiratory and cardiac muscles are affected in DMD, leading to a loss of ambulation, poor respiratory and cardiac function, and a greatly reduced life expectancy due to cardiorespiratory failure^4–6^. Endomysial fibrosis is a hallmark of DMD pathology and an active driver of disease progression as it precedes overt degeneration. Expansion of the interstitial matrix is observed in skeletal muscle biopsies from patients with DMD as young as 2.5 weeks of age^2,7^.

The carefully regulated synthesis and remodelling of the extracellular matrix (ECM) is necessary for effective muscle regeneration. This initially requires the synthesis of a hydrated provisional matrix enriched in versican, hyaluronan and fibronectin, which modulates cell behaviour relevant to inflammation and regeneration^8,9^. This provisional matrix then needs to be carefully remodelled and replaced with a collagen rich, mature matrix^8^. Failed regeneration is characterized by the excessive and persistent accumulation of collagen, proteoglycans and various provisional matrix proteins. Indeed, fibrosis in dystrophic muscles is comprised of mature and provisional matrix proteins, such as the fibrillar collagen isoforms I and III^10,11^, fibronectin^12,13^, and the chondroitin sulphate (CS) proteoglycans decorin, biglycan and the V0/V1 isoforms of versican^14–16^. Excess versican is associated with fibrosis and pathology in lung, liver and cardiovascular disease^17,18^, its role in the pathogenesis of neuromuscular disease is not known. However, versican expression is increased in skeletal muscle biopsies from patients with DMD compared to healthy controls, as assessed by immunohistochemistry^15,19^ and microarray gene expression analysis^20^. Furthermore, in skeletal muscle biopsies from patients with DMD there is far greater deposition of CS/dermatan sulphate side chains than in control biopsies^21^. Versican is likely to be a significant source of these CS side chains, because of all the CS proteoglycans upregulated in dystrophic muscles, V0/V1 versican is the most highly glycosylated^21^.

In skeletal muscle, it is the V0 and V1 isoforms of versican which are most highly expressed^22^. V0/V1 versican is composed of N- and C-terminal globular domains and up to two binding regions for glycosaminoglycan (GAG) CS side chains (GAGα and GAGβ)^23^. The V0 versican variant contains the GAGα and GAGβ domains and is therefore more highly glycosylated than the V1 variant, which only contains the GAGβ domain^24,25^. V0/V1 versican is proteolytically processed by ADAMTS versicanases. This produces the bioactive versikine fragment, which depending on biological context can stimulate apoptosis^26^, inflammation^27^, or modulate mitotic spindle organisation in proliferating cells^28^.

There is emerging evidence that V0/V1 versican synthesis and remodelling is closely associated with cellular processes necessary for effective regenerative myogenesis and driving the pathogenesis of DMD. This includes satellite cell proliferation^29^, myoblast fusion and myofibre formation^22^, and modulation of inflammatory responses^30–32^. By binding cytokines, chemokines and growth factors, such as TGFβ and monocyte chemoattractant protein-1 (MCP-1)^30^, versican CS side chains have important effects on cell signalling and behaviour^23^. TGFβ is highly upregulated in dystrophic muscles, where it is implicated in impaired regenerative myogenesis and fibrosis^33,34^. The pro-fibrotic effects of TGFβ1 include further upregulation of versican synthesis^35^. MCP-1 stimulates monocytes and macrophage infiltration across the vasculature into tissue^30^, and is upregulated in dystrophic muscles^36,37^. Versican also has direct effects on macrophage and monocyte adhesion and migration^38^, and on the monocyte to macrophage transition^39^. Infiltrating macrophages not only synthesise versican, but are also involved in its remodelling through the secretion of ADAMTS versicanases^40^. Fibroblasts are also a source of versican and ADAMTS versicanases^41^. If versican is in excess, then the differentiation of fibroblasts into myofibroblasts is stimulated^42,43^, these are characterized by their high level of collagen synthesis^44^. In *mdx* mice, diaphragm muscles model DMD pathology well, arguably better than hindlimb muscles, presenting with decreased muscle strength and endurance, excessive inflammation, insufficient regeneration, and progressively increasing fibrosis^45–48^. Importantly, V0/V1 versican is highly upregulated^14^.

Metabolic dysfunction is another important sequalae of dystrophin deficiency in patients with DMD and in *mdx* mice^49^. It is characterized by impaired mitochondrial function and reduced ATP production^50^, as well alterations in whole-body metabolism^51^. Physical activity is decreased and energy expenditure is increased in *mdx* mice when compared to wild type mice^51^. Unlike in *mdx* mice, resting energy expenditure is reduced in patients with DMD compared to normal age matched control values^52,53^. Nonetheless, in patients with DMD there is a strong association with resting energy expenditure and vital capacity, with increasing energy requirements as respiratory function declines in the later stages of the disease^52^.

Here, a genetic approach was used to test the hypothesis that versican reduction would ameliorate the pathology of *mdx* mice and improve the function of dystrophic diaphragm muscles. Thus, female *mdx* mice were bred with male heart defect mice (hdf) mice, which are haploinsufficient for the versican allele, to generate F1 *mdx* male pups. At 20–26 weeks of age, male F1 *mdx-*hdf mice with a single functional allele and a concomitant whole-body reduction in versican protein expression and *mdx* control littermates with two functional versican alleles were used to investigate the effects of versican reduction on physical activity, whole-body metabolism, and diaphragm muscle contractile properties and morphology.

## Results

### Full length versican expression is decreased in diaphragm muscles from mdx-hdf mice

**Figure 1:**
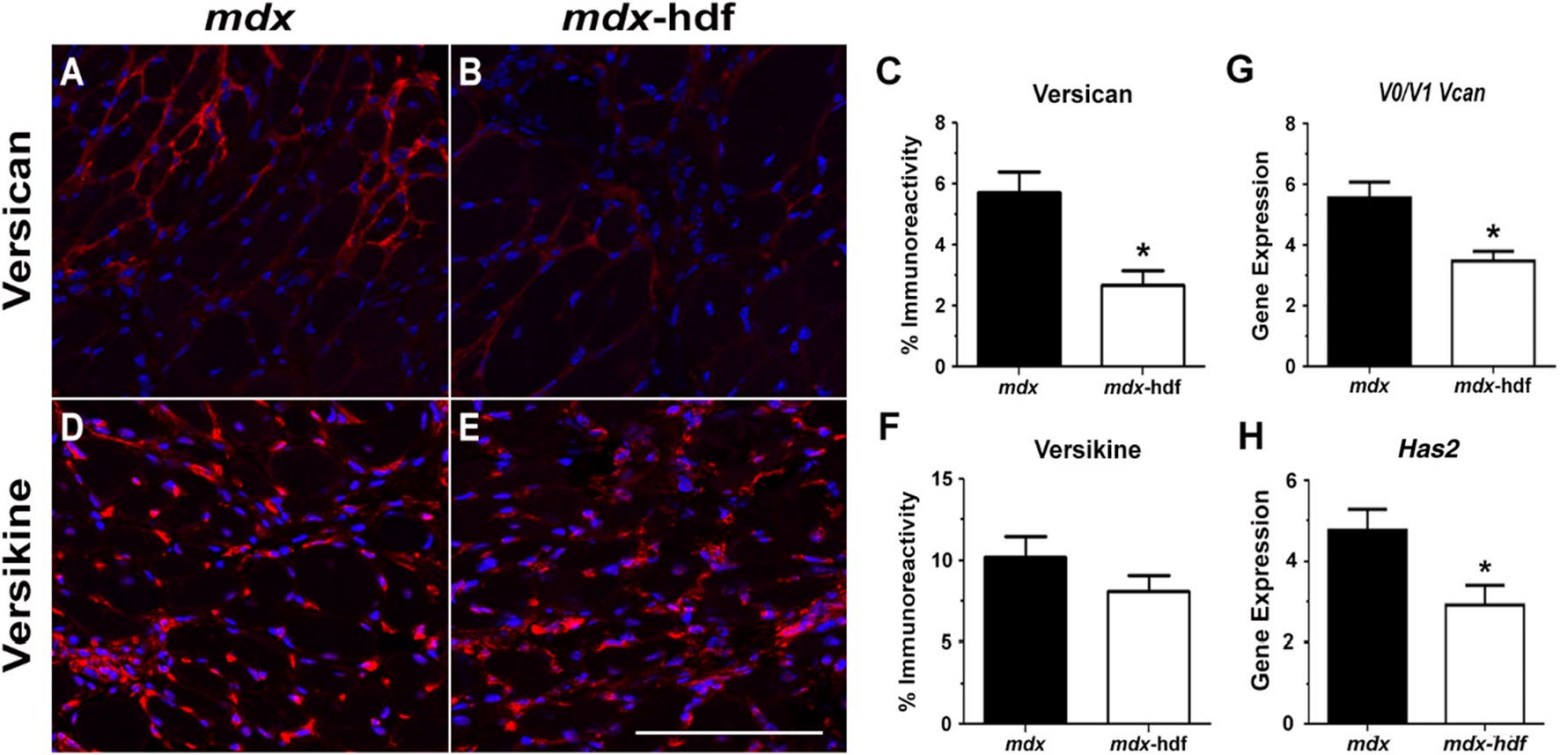
V0/V1 versican and versikine localization and expression in diaphragm muscles from *mdx* and *mdx*-hdf mice. Versican or versikine immunoreactivity in red; nuclei were counterstained with DAPI (blue). (**A, B)** Representative versican staining in diaphragm cross-sections from *mdx* and *mdx*-hdf mice. (**C)** Versican immunoreactivity was reduced in diaphragm muscles from *mdx*-hdf mice compared to *mdx* littermates (^*^p = 0.0004). (**D–E)** Representative versikine staining in diaphragm cross-sections from *mdx* and *mdx*-hdf mice. (**F)** Versikine immunoreactivity did not differ between *mdx* and *mdx*-hdf diaphragm muscles (p = 0.192). (**G)** *V0/V1 Vcan* mRNA transcripts (^*^p = 0.005) and (**H)** *Has2* mRNA transcript (^*^p < 0.05) were reduced in diaphragm muscles from *mdx*-hdf mice versus *mdx* littermates. N = 11–13 *mdx* and N = 11–12 *mdx*-hdf mice for versican and versikine immunoreactivity. N = 12 *mdx* and N = 11 *mdx*-hdf mice for gene expression. Scale bar = 100 µm.

**Figure 2:**
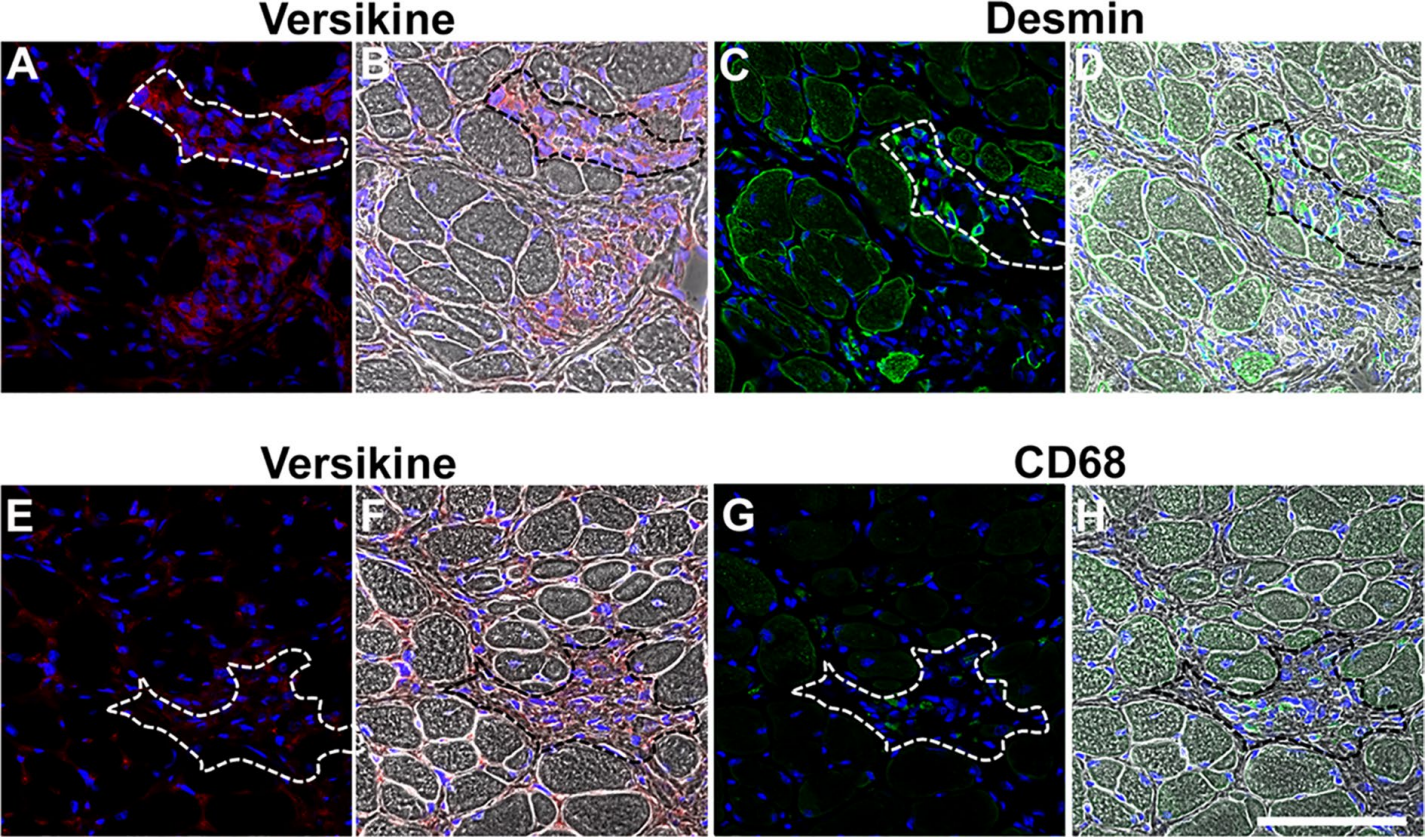
V0/V1 versican remodelling is associated with areas of regeneration and inflammation in *mdx* diaphragm muscles. Serial cross-sections were immunoreacted with primary antibodies against desmin (green) or CD68 (green) and versikine (red); nuclei were counterstained with DAPI (blue). Phase images were captured for orientation. (**A–D)** Desmin positive myoblasts and myotubes or (**E–H)** CD68 positive monocytes and macrophages were localized to regions of versikine immunoreactivity in the endomysium. Scale bar = 100 µm.

**Figure 3:**
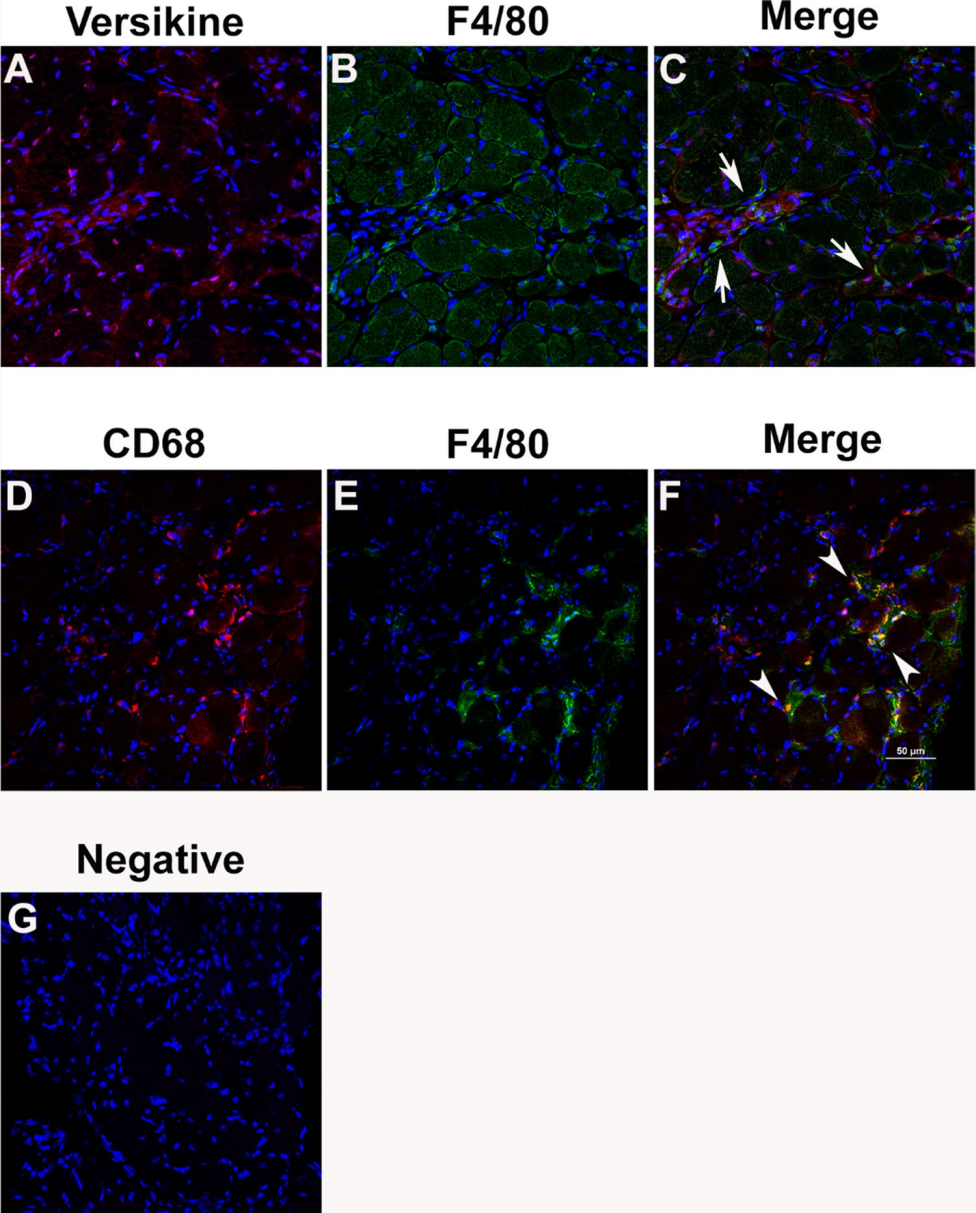
Co-localization of versikine with monocytes and macrophages in *mdx* diaphragm muscles. Diaphragm cross-sections were immunoreacted with primary antibodies against (**A)** versikine (red) and (**B)** F4/80 (green), and nuclei were counterstained with DAP1 to show tissue morphology. (**C)** The merged image shows the presence of F4/80 positive macrophages in regions of versican remodelling (arrows). Diaphragm cross sections were also immunoreacted with primary antibodies against (**D)** CD68 (red) and (**E)** F4/80 (green). (**F)** The colocalization of CD68 with F4/80 (arrowheads) demonstrates the utility of CD68 as an inflammatory cell marker. (**G)** Representative negative control sections reacted with the goat anti-rabbit and goat anti-rat secondary antibodies. Scale bar = 50 µm.

In diaphragm muscle cross-sections from *mdx* and *mdx*-hdf mice, versican immunoreactivity was localised to the endomysium (Fig. 1A, B). Immunoreactivity of the cleaved bioactive versikine fragment was also localised to the endomysium (Fig. 1D, E), as well as to regions of mononuclear infiltrate (Figs. 2A, D, 3A, D), which is comprised of inflammatory cells, myoblasts and fibroblasts. When the percentage of versican immunoreactivity in diaphragm cross-sections was quantified, versican protein expression was reduced by approximately 50% in diaphragm muscles from *mdx*-hdf mice when compared to *mdx* littermates (p = 0.0004; Fig. 1C), which was supported by a similar decrease in *V0/V1 Vcan* mRNA transcript abundance (p = 0.005; Fig. 1G). This confirms successful haploinsufficiency of versican in dystrophic diaphragm muscles using the hdf mouse model. In contrast to full length V0/V1 versican, versikine immunoreactivity did not significantly differ between diaphragm muscles from *mdx* and *mdx*-hdf mice (Fig. 1F). This was not unexpected, as ADAMTS-generated versikine is not an end product and is further degraded^22^. Within the provisional matrix, there is a close association between versican and hyaluronan^9^. Interestingly, the mRNA transcript abundance of *Has2*, the predominant hyaluronan synthase isoform in skeletal muscle^54^, was also decreased in diaphragm muscles from *mdx*-hdf mice compared to *mdx* littermates (p = 0.0329; Fig. 1H).

To demonstrate the association between versican remodelling and regenerative myogenesis or inflammation, serial diaphragm muscle cross-sections were reacted with antibodies against versikine and desmin which is highly expressed in myoblasts and newly regenerated myotubes^55^ or the monocytes and macrophage marker CD68^56,57^. Versikine was highly expressed in regions of mononuclear infiltrate (Fig. 2A, E, white outline), which comprised desmin positive muscle cells (Fig. 2C) and CD68 positive inflammatory cells (Fig. 2G).

To confirm the association between versican remodelling and inflammation, diaphragm cross-sections were reacted with primary antibodies, raised in different species against versikine (Fig. 3A) and the pan-macrophage marker F4/80 (Fig. 3B). In concordance with the serial section findings presented in Fig. 2E–H, F4/80 positive macrophages were co-localized with regions of endomysial versikine staining (Fig. 3C). To confirm the suitability of CD68 as a macrophage marker, diaphragm cross-sections were reacted with the CD68 antibody raised in rabbits (Fig. 3D) and the pan-macrophage marker F4/80 antibody raised in rats (Fig. 3E), as expected co-localization between these two markers was observed (Fig. 3F).

### Effects of versican reduction on the body composition of mdx mice

Despite the association between versican synthesis, myogenesis and muscle growth^29^, the genetic reduction of versican did not affect body weight or composition. Lean mass and fat mass expressed in grams or as a percentage of body weight did not significantly differ between *mdx* and *mdx*-hdf mice (Table 1).

**Table 1:** Body weight, fat mass and lean mass as determined by ECHO-MRI.

	*mdx*	*mdx*-hdf	P value
Body weight (g)	32.24 ± 0.50	32.46 ± 0.70	0.80
Fat (g)	1.44 ± 0.20	1.78 ± 0.15	0.18
Fat (%)	4.41 ± 0.57	5.49 ± 0.44	0.14
Lean mass (g)	29.77 ± 0.41	30.22 ± 0.66	0.58
Lean mass (%)	92.40 ± 0.52	93.10 ± 0.68	0.43

Data are mean ± SEM.

N = 14 *mdx* and N = 16 *mdx*-hdf mice.

### Increased spontaneous physical activity in mdx-hdf mice

**Figure 4:**
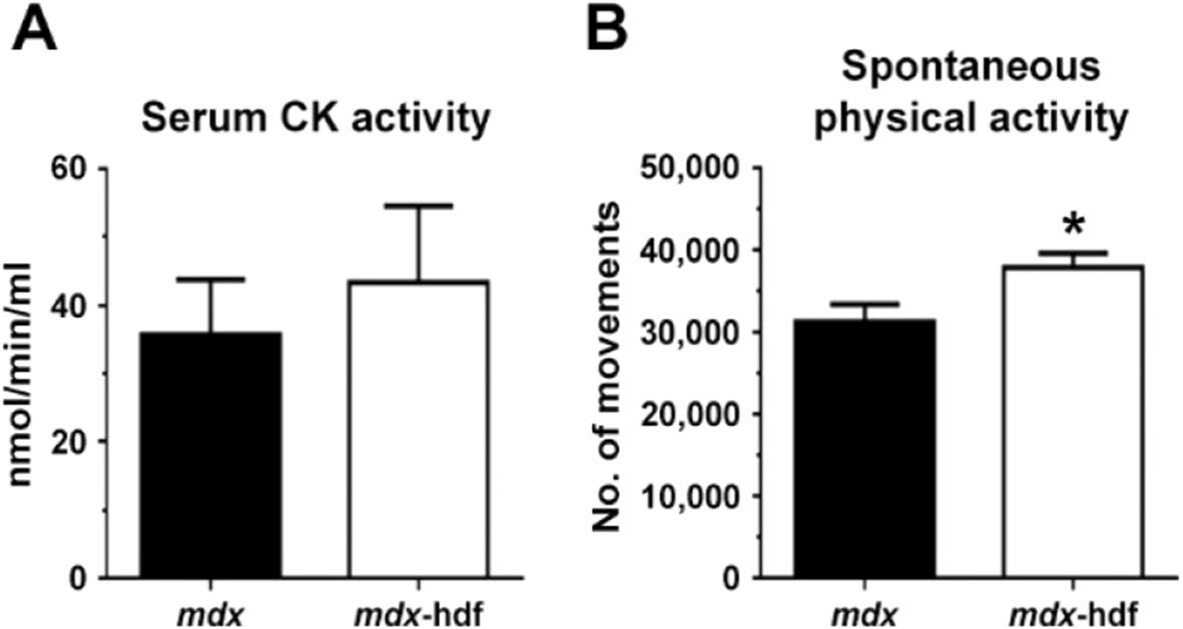
The genetic reduction of versican was associated with increased spontaneous physical activity. (**A)** Serum CK activity did not significantly differ between *mdx* and *mdx*-hdf mice (p = 0.598). (**B)** During 24 h in metabolic cages, *mdx*-hdf mice were more active than their *mdx* littermates as indicated by an increase in total movement (^*^p = 0.024). N = 8 *mdx* and N = 9 *mdx*-hdf for serum CK activity, and N = 12 *mdx* and N = 15 *mdx*-hdf mice for spontaneous physical activity analysis.

Serum creatine kinase (CK) activity, a marker of muscle damage, was not significantly affected by the genetic reduction of versican (Fig. 4A), despite the *mdx*-hdf mice being more physically active than their *mdx* littermates (p = 0.024; Fig. 4B). This increase in spontaneous physical activity in *mdx*-hdf mice is interesting given that physical activity and exercise capacity are reduced in *mdx* mice compared to wild type mice^51^.

### The genetic reduction of versican has favourable effects on whole-body energy balance and metabolism in mdx mice

**Figure 5:**
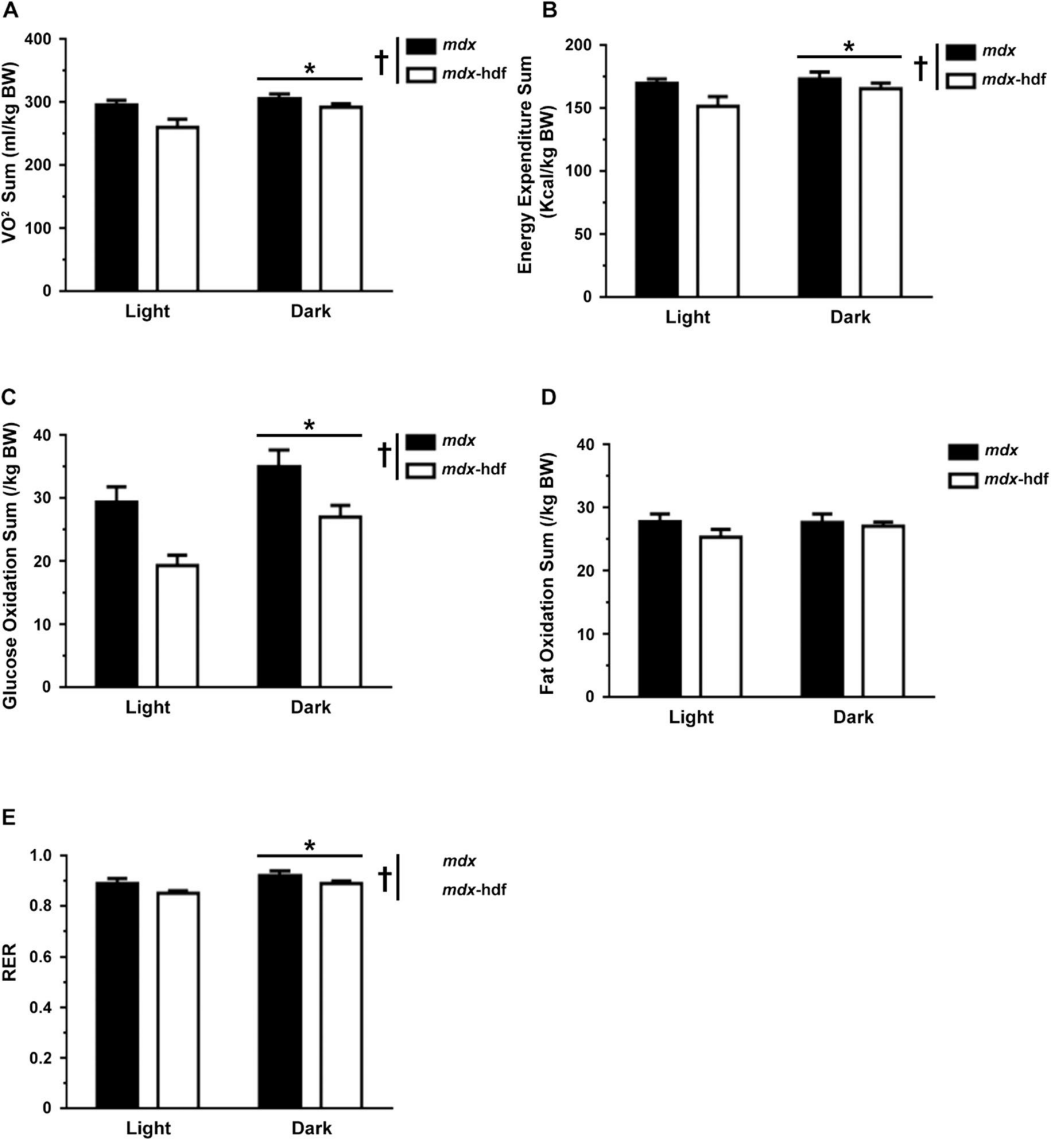
The genetic reduction of versican improved whole-body energy balance in *mdx* mice. (**A)** ^*^ In all mice, oxygen consumption (VO_2_ 12 h sum) was higher during the 12 h dark period versus the 12 h light period. ^†^ Irrespective of the light or dark period, VO_2_ sum was lower in *mdx*-hdf mice compared to *mdx* littermates. (**B)** ^*^ Energy expenditure (12 h sum) was higher during the 12 h dark period and ^†^ lower in *mdx*-hdf mice. (**C)** ^*^ Glucose oxidation (sum) was higher during the 12 h dark period and ^†^ lower in *mdx*-hdf mice. (**D)** There was no significant difference in lipid oxidation (12 h sum) between the dark and light period nor between *mdx* and *mdx*-hdf mice. (**E)** ^*^ RER was higher during the 12 h dark period and ^†^ lower in *mdx*-hdf mice. † P < 0.05; main effect genotype; 2-way GLM-ANOVA; ^*^p < 0.05; main effect time; 2-way GLM-ANOVA). N = 12 *mdx* and N = 15 *mdx*-hdf mice.

Corresponding to increased nocturnal activity, in *mdx*-hdf mice and *mdx* littermates, oxygen consumption (VO_2_ 12 h sum), energy expenditure (12 h sum), glucose oxidation (12 h sum), and the respiratory exchange ratio (RER), but not fat oxidation (12 h sum), were higher during the 12 h dark than the 12 h light period (p < 0.05; Fig. 5A–C, E). Average 12 h values for oxygen consumption (VO_2_), energy expenditure, glucose and fat oxidation were also calculated, and similar diurnal trends were observed (data not shown).

The genetic reduction of versican may have favourable effects on whole-body metabolism. When summed over the 12 h dark or light period, total oxygen consumption (VO_2_ 12 h sum) (p = 0.006; Fig. 5A), exergy expenditure (p = 0.033; Fig. 5B), glucose oxidation (p < 0.001; Fig. 5C), but not fat oxidation (Fig. 5D), were lower in *mdx*-hdf mice compared to *mdx* littermates. In concordance with the reduction in glucose oxidation, the 12 h average RER values were lower in *mdx*-hdf mice compared to *mdx* littermates (p = 0.013; Fig. 5E), irrespective of the light or dark period.

### Stroke volume is maintained despite increased heart mass and left ventricular dilatation in mdx-hdf mice

Similar to patients with DMD, older *mdx* mice develop a dilated cardiac myopathy and myocardial fibrosis which can be observed from 42 weeks of age^58^. Hdf mice were first used to establish that versican is essential for the correct embryonic development of the heart, including formation of the right ventricle and the intraventricular septum^59–61^. Given that versican is also associated with fibrosis in various pathological contexts^9^, echocardiography was used to assess the morphological and functional effects of versican reduction on the *mdx* heart. At 25 weeks of age, there was no histological evidence of fibrosis in hearts from either *mdx* or *mdx*-hdf mice (Fig. S1). However, hearts from *mdx*-hdf mice were heavier than those from *mdx* littermates (p = 0.032; Table 2), even when normalized to body weight (p = 0.020; Table 2). Based on echocardiography measures, the thickness of the intraventricular septum (IVS) and the left ventricular posterior wall (LVPW) dimensions did not significantly differ between *mdx* and *mdx*-hdf mice in either systole or diastole. During systole, but not diastole, the left ventricular internal diameter (LVID) was greater in *mdx*-hdf mice compared to *mdx* littermates (p = 0.03). This suggests development of a dilated cardiac myopathy with systolic dysfunction. In concordance with this, fractional shortening (FS; p = 0.02) and the ejection fraction (EF; p = 0.03) were decreased in hearts from *mdx*-hdf mice. Nonetheless, stroke volume was maintained in *mdx*-hdf mice, such that it did not differ from *mdx* littermates.

**Table 2:** Echocardiography findings from *mdx* and *mdx*-hdf mice at 25 weeks of age.

	**Units**	***mdx***	***mdx-hdf***	**P value**
Heart weight	mg	142.6 ± 3.0	153.0 ± 3.4	0.03*
Heart:Body weight ratio	mg:g	4.4 ± 0.1	4.7 ± 0.1	0.02*
IVSd (MM)	cm	0.099 ± 0.004	0.105 ± 0.002	0.15
IVSs (MM)	cm	0.185 ± 0.006	0.178 ± 0.010	0.58
LVIDd (MM)	cm	0.284 ± 0.009	0.309 ± 0.020	0.35
LVIDs (MM)	cm	0.122 ± 0.011	0.173 ± 0.015	0.03*
LVPWDd (MM)	cm	0.106 ± 0.005	0.106 ± 0.005	0.95
LVPWDs (MM)	cm	0.159 ± 0.006	0.152 ± 0.007	0.43
SV (MM-cubed	ml	0.021 ± 0.002	0.027 ± 0.005	0.36
EF (MM-cubed)	%	90.5 ± 2.5	80.7 ± 3.0	0.03*
FS (MM-cubed	%	56.8 ± 4.0	44.1 ± 3.2	0.02*
LV Mass (cubed)	g	0.678 ± 0.004	0.697 ± 0.011	0.20

Data are mean ± SEM.

IVS d/s = interventricular septal dimension (diastole/systole); LVID d/s = left ventricle internal diameter (diastole/systole); LVPWD d/s = left ventricular posterior wall dimensions (diastole/systole); SV = stroke volume; EF = ejection fraction; FS = fractional shortening; LV = left ventricle.

N = 13 *mdx* and N = 16 *mdx*-hdf for heart weight data. N = 8 *mdx* and N = 12 *mdx*-hdf for echocardiography.

### The genetic reduction of versican improved the ex-vivo strength and endurance of dystrophic diaphragm muscles

In response to a 1 Hz stimulation, a modest increased in time to peak tension (TPT) was observed in diaphragm muscles from *mdx*-hdf mice (p = 0.03), whilst normalized twitch force (sP_t_) and half relaxation time (½RT) were not significantly increased in diaphragm muscle strips from *mdx*-hdf mice versus *mdx* littermates (Table 3).

**Table 3:** *Ex-vivo* twitch (1 Hz) contractile properties of diaphragm muscles from *mdx* and *mdx*-hdf at 21 weeks of age.

	Units	*mdx*	*mdx-*hdf	P value
Diaphragm strip mass	mg	8.18 ± 0.57	7.95 ± 0.55	0.77
sP_t_	kN/m^2^	14.45 ± 1.80	17.97 ± 1.64	0.17
TPT	s	0.2301 ± 0.0005	0.2314 ± 0.0004	0.03*
½ RT	s	0.031 ± 0.002	0.035 ± 0.002	0.11

Data are mean ± SEM.

sP_t_ = specific twitch force; TPT = time to peak tension; ½ RT = half relaxation time.

N = 11 *mdx* mice and N = 15 *mdx*-hdf mice.

**Figure 6:**
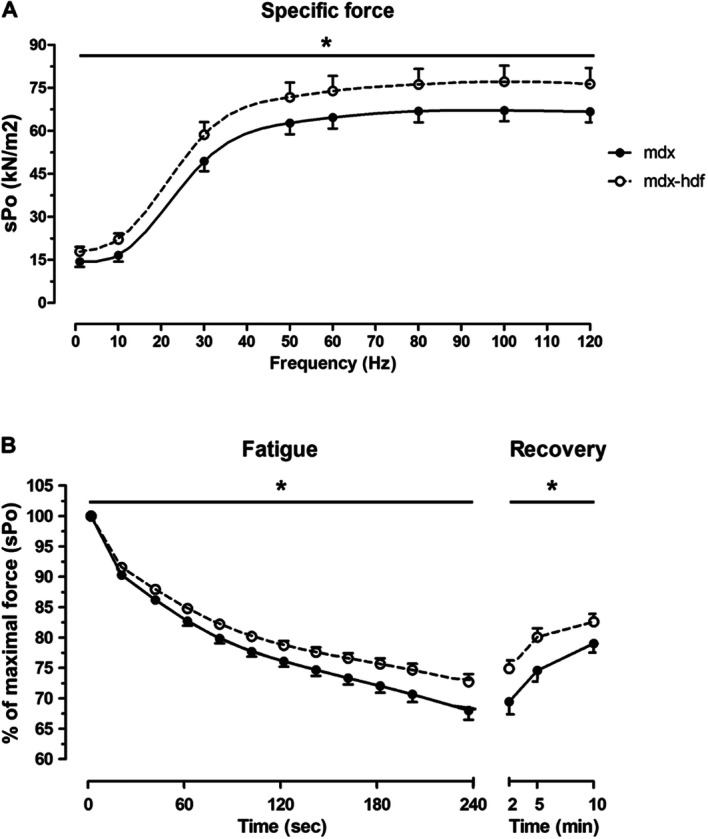
The genetic reduction of versican increased the ex vivo strength and endurance of isolated costal diaphragm muscle strips. (**A)** Haploinsufficiency of versican increased the specific force output (sP_o_) of diaphragm muscle strips, as indicated by the upward shift in the force frequency curve (^*^p < 0.001; main effect genotype; 2-way GLM-ANOVA). (**B)** During 4 min of intermittent, 60 Hz stimulation, the relative fatigability of diaphragm muscles from *mdx*-hdf mice was reduced versus *mdx* littermates (*p < 0.001; main effect genotype; 2-way GLM ANOVA), and following 2, 5 and 10 min of rest relative force recovery was also improved (*p < 0.001; main effect genotype; 2-way GLM ANOVA). N = 11 *mdx* and N = 14 *mdx*-hdf mice.

With increasing stimulation frequency, the genetic reduction of versican increased the normalized force output (sP_o_) of diaphragm muscles, as indicated by an upward shift of the force frequency curve (p < 0.001; Fig. 6A). To assess fatigability and force recovery, diaphragm muscle strips were subjected to 4 min of intermittent, 60 Hz stimulation with force recovery assessed at 2, 5 and 10 min post fatigue. During the 4 min fatigue protocol, diaphragm muscles from *mdx*-hdf mice fatigued less compared to their *mdx* littermates (p < 0.001), and force recovery was also improved (p < 0.001; Fig. 6B).

### Muscle morphology and gene markers of myogenesis and apoptosis in dystrophic diaphragm muscles in response to the genetic reduction of versican

**Figure 7:**
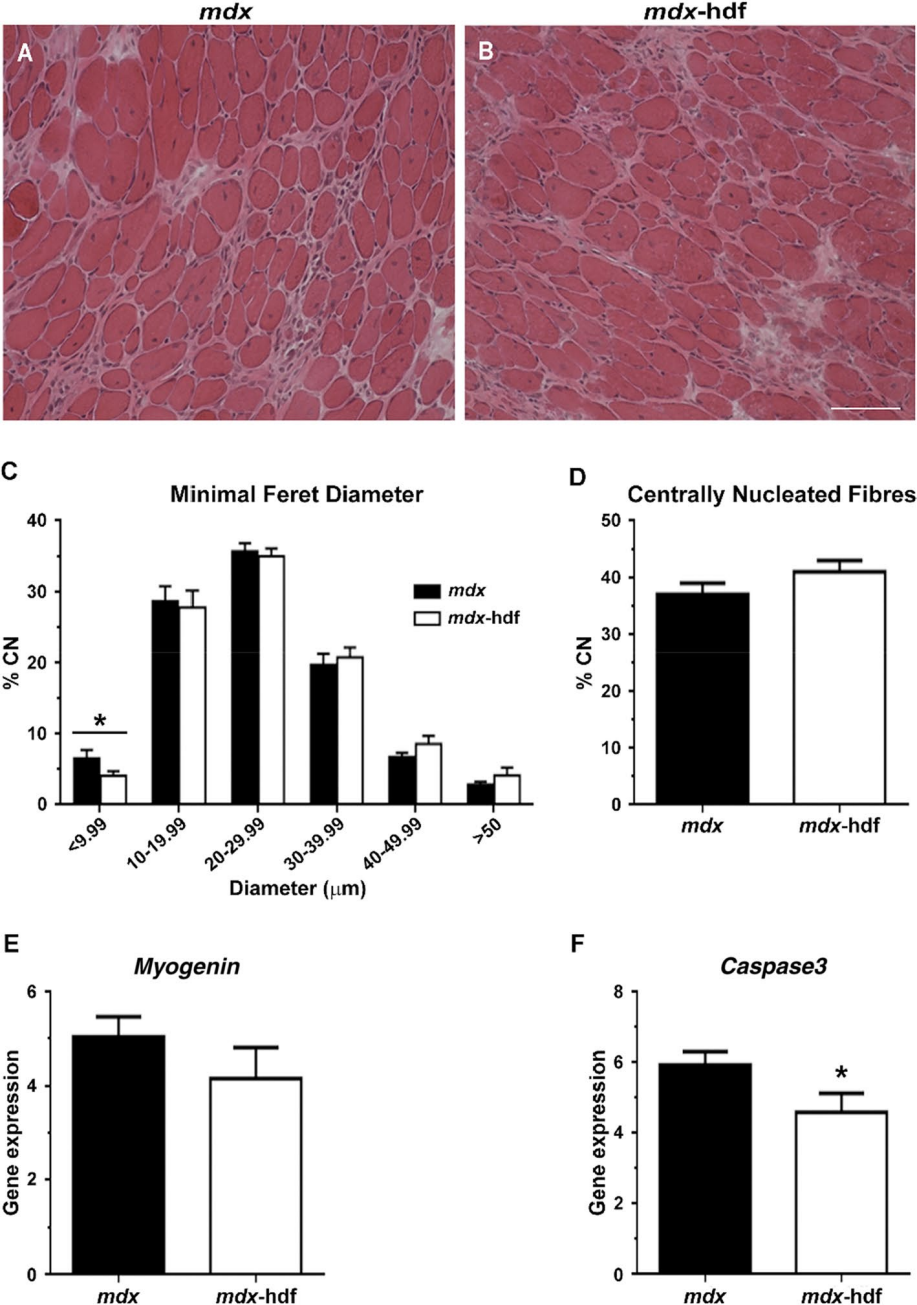
Effects of versican reduction diaphragm muscle morphology and gene markers of myogenesis and apoptosis. (**A–B)** Representative H&E stained sections of *mdx* and *mdx*-hdf diaphragm cross-sections. (**C)** Diaphragm muscles from *mdx*-hdf had fewer very small fibres > 9.99 µm in diameter (^*^p = 0.031; *t* test). (**D)** The percentage of centrally nucleated fibres did not significantly differ between muscles from *mdx*-hdf mice versus *mdx* littermates. (**E)** The mRNA transcript abundance of *Myogenin* did not significantly differ between diaphragm muscles from *mdx*-hdf mice and *mdx* littermates (p = 0.2575). (**F)** The mRNA transcript abundance of *Caspase 3* tended to be lower in diaphragm muscles from *mdx*-hdf mice (p = 0.055). N = 11 *mdx* and N = 11 *mdx*-hdf mice for muscle histology and morphology. N = 12 *mdx* and N = 12 *mdx*-hdf mice for gene expression analysis. Scale bar = 100 µm*.*

Characteristic of the pathology of dystrophic diaphragm muscles, myofibres were variable in size and greater than one third were centrally nucleated, indicative of damage and repair. In concordance with V0/V1 versican and verskine immunoreactivity results (Figs. 1, 2), endomysial fibrosis and mononuclear infiltrate were also readily evident in H&E strained cross-sections (Fig. 7A, B). With the exception that diaphragm muscles from *mdx*-hdf mice had significantly fewer very small myofibres < 9.99 µm in size (p = 0.031; Fig. 7C), the genetic reduction of versican had negligible effects on muscle fibre size, as assessed using minimal feret diameter. With regards to markers of muscle regeneration, the genetic reduction of versican did not significantly alter the proportion of centrally nucleated fibres (Fig. 7D) nor did it alter the mRNA transcript abundance of *Myogenin* (Fig. 7E). *Caspase-3* gene expression tended to be lower in diaphragm muscles from *mdx*-hdf mice compared to *mdx* littermates (p = 0.055; Fig. 7F), whether this is in fact associated with decreased apoptosis and degeneration requires further investigation, as this too could contribute to the positive effects of versican reduction on *mdx* diaphragm muscle function (Fig. 6).

### Inflammation and fibrosis in dystrophic diaphragm muscles in response to the genetic reduction of versican

**Figure 8:**
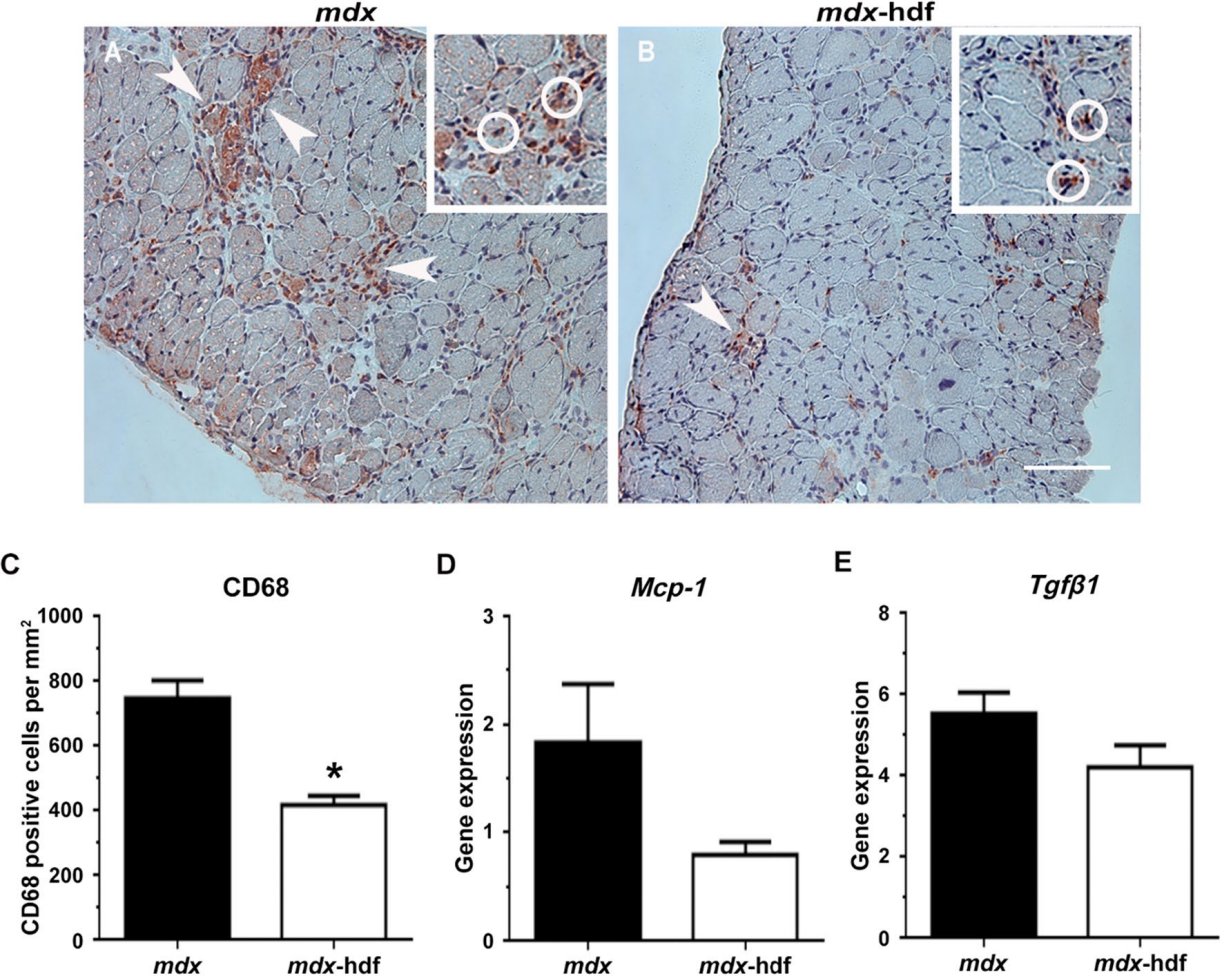
Versican reduction decreased inflammation in dystrophic diaphragm muscles. (**A–B)** Representative CD68 staining in diaphragm cross-sections from *mdx* and *mdx*-hdf mice with CD68 positive macrophages (brownish-red; AEC) and nuclei (blue; hematoxylin). (**C)** The number of infiltrating CD68 positive monocytes or macrophages per mm^2^ tissue was lower in diaphragm cross-sections from *mdx*-hdf mice compared to *mdx* littermates (^*^p < 0.0001). (**D–E)** *Mcp-1* and *Tgfβ1* mRNA transcript abundance was not significantly decreased in diaphragm muscles from *mdx*-hdf mice versus *mdx* littermates (p = 0.085 and p = 0.096; respectively). N = 10 *mdx* and N = 15 *mdx*-hdf mice for assessment of macrophage infiltration. N = 12 *mdx* and N = 12 *mdx*-hdf mice for gene expression analysis. Scale bar = 100 µm*.*

The genetic reduction of versican attenuated inflammation in dystrophic diaphragm muscles, as indicated by an approximately 50% decrease in the number of infiltrating CD68 positive macrophages and monocytes in diaphragm muscle cross-sections from *mdx*-hdf mice versus *mdx* littermates (p < 0.001; Fig. 8A–C). This is in concordance with published observations, that excess V0/V1 versican stimulates macrophage infiltration in various pathological contexts^62,63^. Furthermore, the mRNA transcript abundance of the inflammatory markers *Mcp-1* and *Tgfβ1* mRNA tended to be decreased in diaphragm muscles from *mdx*-hdf mice (p = 0.085 and p = 0.096, respectively; Fig. 8D–E).

**Figure 9:**
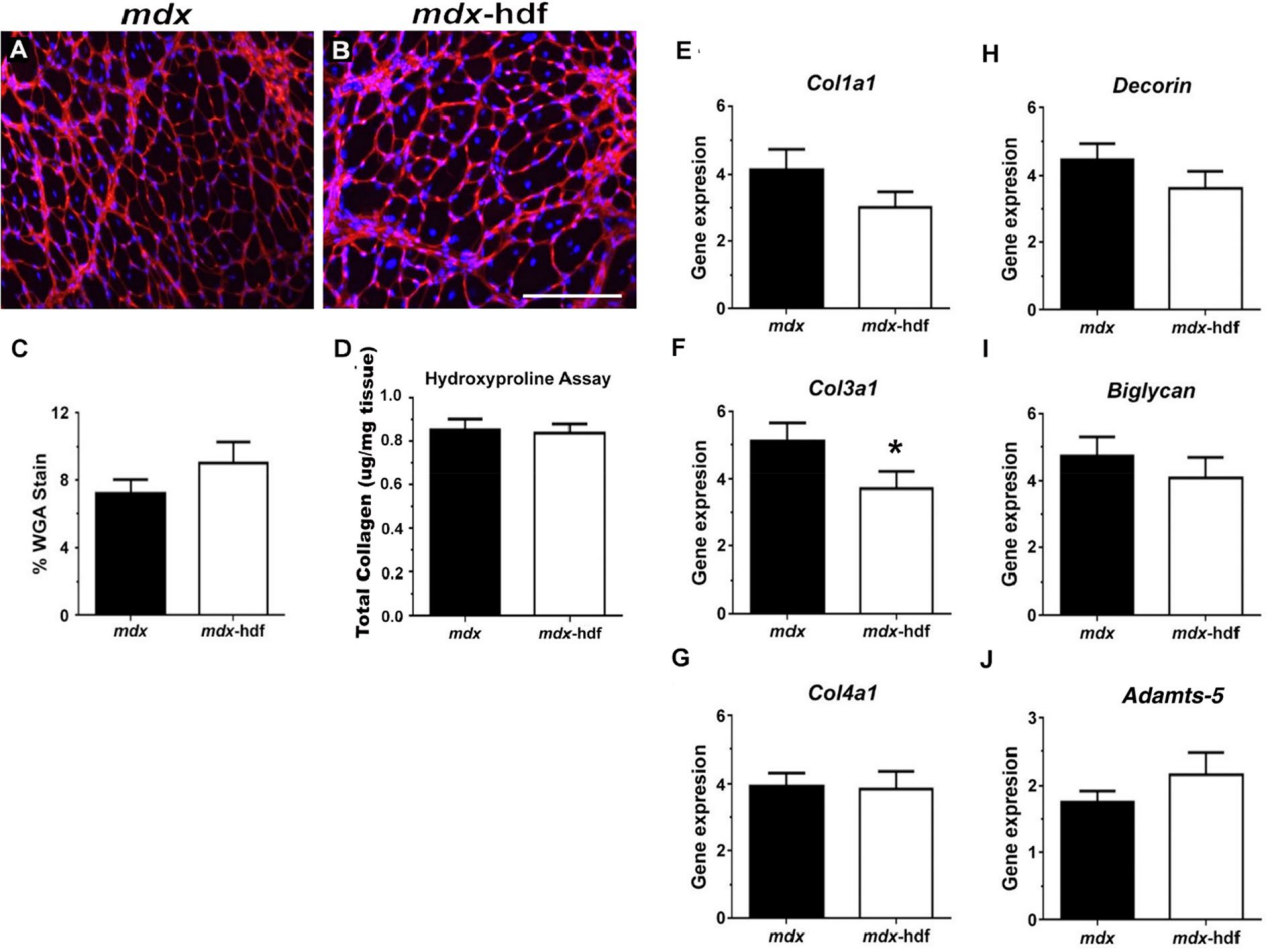
Versican reduction does not attenuate fibrosis in dystrophic diaphragm muscles. (**A–B)** Representative *mdx* and *mdx*-hdf diaphragm sections stained with WGA (red) as marker of fibrosis; nuclei (blue; DAP1). (**C)** Fibrosis, as quantified by the proportion (%) of muscle cross-section stained with WGA, did not differ between the diaphragm muscles from *mdx* and *mdx*-hdf mice (p = 0.251). (**D)** Collagen content, as assessed by the hydroxyproline assay, also did not significantly differ between diaphragm muscles from *mdx* and *mdx-*hdf mice (p = 0.820). (**E–K)** With the exception of *Col3a1* (^*^p = 0.033), versican reduction did not significantly decrease the mRNA transcript abundance of representative ECM proteins; specifically *Col1a1* (p = 0.082), *Col4a1* (p = 0.390), *Decorin* (p = 0.083), *Biglycan* (p = 0.298), and *Adamts-5* (p = 0.076). N = 9 *mdx* and  N = 10 *mdx*-hdf mice assessment of fibrosis with WGA. N = 8 *mdx* and N = 10 *mdx*-hdf mice for the hydroxyproline assay. N = 12 *mdx* and N = 12 *mdx*-hdf mice for gene expression analysis. Scale bar = 200 µm*.*

By binding to proteoglycans^64^ and collagen^65^, which are all upregulated in dystrophic muscles^3,66^, WGA can be used to visualize and quantify fibrosis^67^. Despite the reduction in versican expression, the proportion of muscle cross-section stained with WGA did not differ between diaphragm muscle cross-sections from *mdx* and *mdx*-hdf mice (Fig. 9C). To assess whether targeting the provisional matrix would affect the deposition of a collagen rich mature matrix^8^, hydroxyproline content, a measure of tissue collagen content^68^, was determined in diaphragm muscle lysates from *mdx*-hdf mice and *mdx* littermates. In concordance with the WGA data, the genetic reduction of versican did not significantly alter the collagen content of dystrophic diaphragm muscles (Fig. 9D). With the exception of *Col3a1* (p = 0.033), *Col1a1* (p = 0.082), *Col4a1* (p = 0.390), *Decorin* (p = 0.083), *Biglycan* (p = 0.298), and *Adamts-5* (p = 0.076) gene expression was not significantly different in *mdx*-hdf mice compared to *mdx* littermates (Fig. 9E–K). Thus, in dystrophic diaphragm muscles the genetic reduction of versican had very modest effects on the transcription of ECM associated genes which were not supported by histological and biochemical markers of fibrosis.

### The effects of versican reduction on fibre type in dystrophic diaphragm muscles

**Figure 10:**
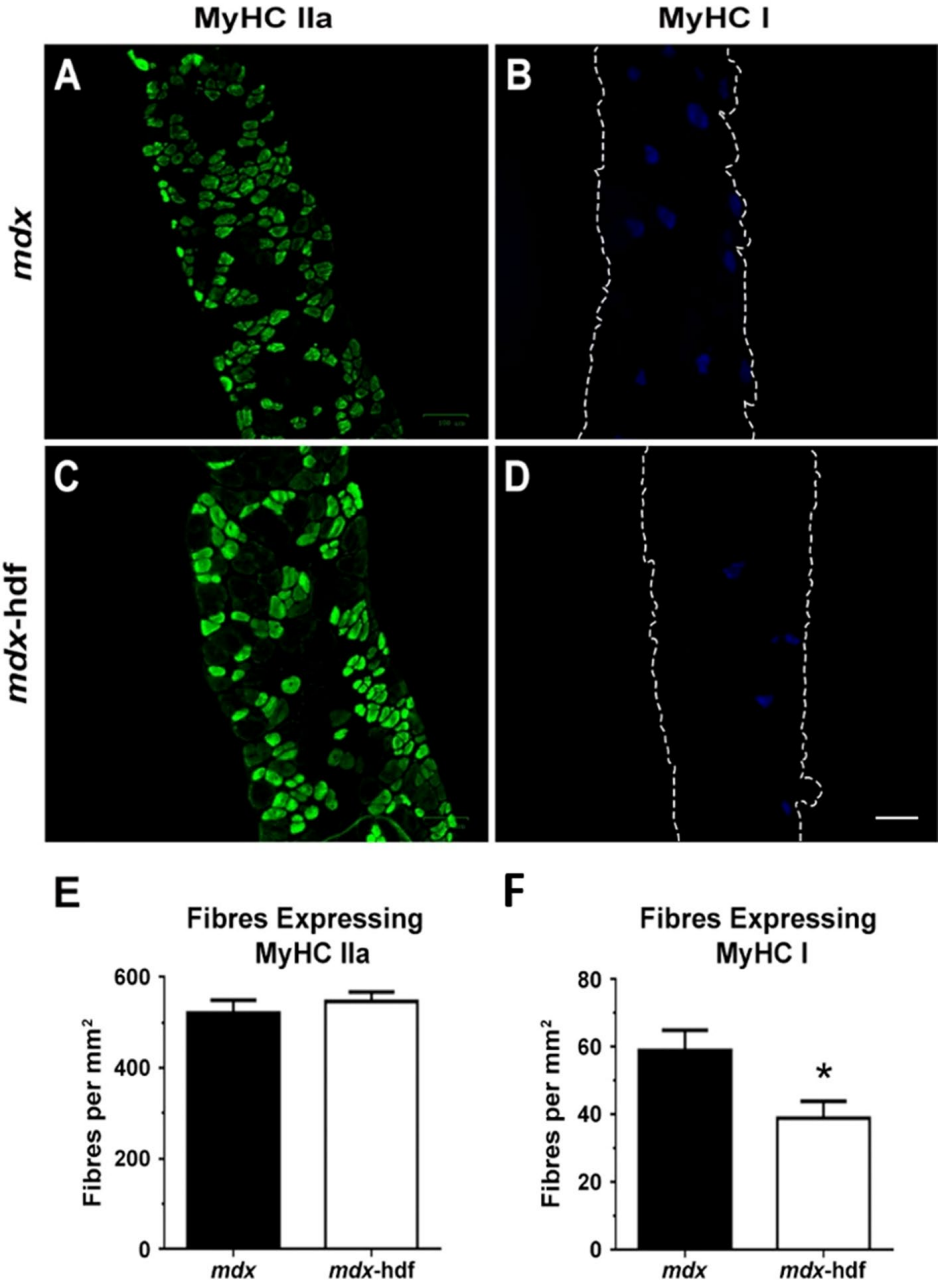
Effects of versican reduction on myosin heavy chain (MyHC) isoform expression in dystrophic diaphragm muscles**. (A–D)** Representative cross-sections of MyHC type IIa (green) and MyHC type I (blue) immunoreactivity. (**E)** The number of MyHC type IIa fibres per mm^2^ of diaphragm cross-section did not significantly differ between *mdx* and *mdx*-hdf mice (p = 0.4516; *t* test). (**F)** The number of MyHC type I fibres per mm^2^ of diaphragm cross-section was reduced in *mdx*-hdf mice (*p = 0.0215). N = 10–11 *mdx* and N = 10 *mdx*-hdf mice. Scale bar = 100 µm.

The predominant myosin heavy chain (MyHC) isoform in diaphragm muscles from adult *mdx* mice is the fast oxidative MyHC type IIa isoform, with 55% fibres expressing this isoform whilst 10% of fibres express the slow MyHC type I^69^. This is in concordance with our observations of a greater prevalence of MyHC type IIa than MyHC type I fibres in dystrophic diaphragm muscle cross-sections (Fig. 8A–D). The number of MyHC type IIa fibres per mm^2^ of diaphragm cross-section did not significantly differ between *mdx* and *mdx*-hdf mice (p = 0.4516; Fig. 10E). However, the number of MyHC type I per mm^2^ of diaphragm cross-section was ~ 30% lower in *mdx*-hdf mice compared to *mdx* littermates (^*^p = 0.0215; Fig. 10F). This reduction in the proportion of MyHC type I fibres, highlights the complex biological effects of versican in dystrophic muscles and may have have implications for contractile function.

## Discussion

V0/V1 versican is highly upregulated in muscle biopsies from patients with DMD^15,19^ and in dystrophic diaphragm muscles from *mdx* mice^14^. Here, we present initial evidence that aberrant versican synthesis and remodelling may contribute to skeletal muscle dysfunction and degeneration in dystrophy. Specifically, we confirm the higher expression levels of versican in diaphragm muscles from *mdx* mice and show that the bioactive versikine fragment is co-localized with macrophages and monocytes (as identified by their CD68 and F4/80 immunoreactivity), as well as desmin positive myoblasts and newly regenerative myofibres. This highlights the association between versican remodelling, inflammation and myogenesis in dystrophic diaphragm muscles.

Importantly, when V0/V1 versican gene and protein expression was reduced by approximately 50% in diaphragm muscles from *mdx* mice, ex vivo strength and endurance was improved, and inflammation (specifically, monocyte and macrophage infiltration) was attenuated. These improvements in diaphragm muscle contractile function and pathology were associated with an increase in spontaneous physical activity in *mdx-*hdf mice. Despite this increase in physical activity, there was no associated increase in muscle damage, as determined by serum CK activity and morphometric analysis of diaphragm muscle cross-sections, in *mdx-*hdf mice. Furthermore, the genetic reduction of versican had positive effects on whole-body energy balance and metabolism, as indicated by the decrease in whole-body oxygen consumption (VO_2_), energy expenditure and glucose oxidation. This is an important observation as *mdx* mice are less active and have a higher energy expenditure than control, wild type C57/BL10 mice^51^. This high energy demand of dystrophy is also observed in young steroid-naive boys with DMD and manifests itself as compromised growth, height specifically, from a very early age^70^. Altogether, our findings demonstrate the potential of targeting dysregulated versican synthesis to ameliorate the pathology of dystrophy.

This association between versican and inflammation has been reported in other disease contexts, where versican is thought to generate a matrix which promotes leukocyte migration and adhesion^41,71^. Monocytes and macrophages can synthesise^72^ and remodel V1/V0 versican^73,74^. In other biological contexts, a higher level of versican expression is observed in pro-inflammatory M1 compared to anti-inflammatory M2 macrophages^72^. This is interesting as in *mdx* mice the balance between M1 and M2 macrophages influences regenerative myogenesis with an excess of M1 macrophages being detrimental to dystrophic muscles^75–77^. Recently, Coles et al*.* proposed that the ECM may be a major source of pro-inflammatory molecules which potentiate the immune response and drive pathology in dystrophic muscles, and they highlight versican as one such matrix protein^19^. This proposition is supported by our findings where the genetic reduction of versican decreased infiltration of CD68 positive macrophages and monocytes into diaphragm muscles from *mdx*-hdf mice compared to *mdx* littermates. In follow-up studies, it would be interesting to investigate whether versican reduction affects not only inflammatory cell infiltration, but also macrophage phenotype and  polarization. Corroborating this phenotype of reduced inflammation is the trend towards decreased *Mcp-1* and *Tgfβ1* gene expression. We would argue that this reduction in inflammation in *mdx*-hdf mice may have contributed to the increase in physical activity and the improvement in fatigability of isolated diaphragm muscle strips. Indeed, when pharmacological strategies, such as glucocorticoid treatment^78^, protein kinase C θ inhibition^79^, or blockade of the Il-6 receptor^80^ were used to attenuate inflammation in *mdx* mice, treadmill running performance, specifically the time to exhaustion, was improved.

The genetic reduction of versican also improved the ex vivo force output of dystrophic diaphragm muscles. Contributing factors may include decreased inflammatory cell infiltration^81^. Monocytes and macrophages, especially M1 macrophages, are a potent source of pro-inflammatory mediators such as tumour necrosis factor-α (TNFα). These exacerbate pathology and potentiate contractile dysfunction in dystrophic muscles. In *mdx* mice, the genetic deletion of TNFα improved the ventilatory function, including increased ex vivo strength (sP_o_) of diaphragm muscle strips^82^. Similar to our observations, this was associated with reduced expression of the MyHC type I isoform and no significant change in the MyHC type IIa isoform^82^. Whether the decrease in monocyte and macrophage infiltration in diaphragm muscles from *mdx*-hdf mice was associated with a concurrent reduction in TNFα protein levels should be investigated in follow-up studies.

The reduction in the proportion of very small muscle fibres (< 9.99 μm in diameter) in diaphragm muscles from *mdx*-hdf mice may be another contributing factor to the increase in force producing capacity. The positive correlation between fibre size and strength is well established^83^, and the force producing capacity of these very small fibres is likely to be quite limited. Whilst the mechanisms as to why versican reduction affected the proportion of these very small muscle fibre size remain to be elucidated. Fewer very small fibers may relate to changes in de novo fiber formation and regeneration, and perhaps improved regenerative myogenesis. The upregulation of V0/V1 versican in dystrophic diaphragm muscles may impair fibre growth during regenerative myogenesis. Indeed, in differentiating C2C12 myoblasts inadequate clearance of a versican-rich provisional matrix leads to impaired myoblast fusion and myotube formation^22^. Similarly, excess CS chains in the pericellular and interstitial matrix reduce myoblast fusion and myofibre growth in vitro and in vivo^84^, as V0/V1 versican is highly glycosylated and the genetic reduction of versican should reduce CS abundance in dystrophic muscles. This proposition needs to be carefully interrogated in follow-up studies using immunohistochemical staining for the embryonic and neonatal MyHC isoforms, which are expressed following the initiation of regeneration at 1 to 3 days post-damage and during ongoing regeneration 1 to 3 weeks post injury, respectively. It would be important to assess not just the proportion of embryonic and neonatal MyHC isoform positive fibres, but also their respective fibres size (min feret diameter).

It is unlikely that the increase in the specific force output of diaphragm muscles from *mdx*-hdf mice was mediated by a reduction of fibrosis. The genetic reduction of versican, had very modest effects on ECM gene markers. With the exception of *Has2* and *Col3a1,* the mRNA transcript abundance of *Adamts-5*, *Col1a1, Col4a1,* and the proteoglycans *Biglycan* and *Decorin* did not differ between diaphragm muscles from *mdx*-hdf and *mdx* mice. The gene data are supported by histological and biochemical analyses of fibrosis using WGA and the hydroxyproline assay (as a measure of total collagen content). In designing this study, we had  hypothesized that versican reduction would attenuate fibrosis in diaphragm muscles from *mdx* mice given the potential bidirectional association between versican and fibrosis in other pathological contexts. For example, liver fibrosis is associated with excess versican synthesis, and in cultured hepatic stellate cells versican knockdown inhibited the expression of fibrogenic genes including *Tgfβ1* and *Collagen 1*^17^. TGFβ is a major driver of fibrosis in dystrophic muscles^3,34^. Versican can regulate TGFβ bioavailability and increase active signalling in other biological contexts^42^, whether TGFβ signaling was altered by versican reduction in *mdx*-hdf mice remains to be determined and as this may contribute to the lack of effect of versican reduction on fibrosis.

The effects of versican reduction on whole-body metabolism and diaphragm muscle endurance were unexpected, and it is unlikely that decreased inflammation is the only underpinning mechanism. Worthy of further investigation are the potential interactions between a versican-rich extracellular matrix and mitochondrial function, as there is increasing recognition that carefully regulated ECM synthesis and remodeling is fundamental for metabolic regulation^85,86^. In hepatocellular carcinoma cells and patients with hepatocellular carcinoma, increased V0 versican stimulated glucose uptake and aerobic glycolysis^87^. In cultured vascular endothelial cells, excess versican induced mitochondrial dysfunction when transported by exosomes to vascular smooth muscle cells^88^. Whilst, in cell culture models of axon growth, chondroitin sulphate proteoglycans, though not versican specifically, impaired mitochondrial respiration and decreased ATP synthesis through downstream deleterious effects on the mitochondrial membrane potential, mitochondrial biogenesis and morphology^89,90^. In the context of insulin resistance and diabetes, aberrant remodelling of the skeletal muscle extracellular matrix alters mechano-signal transduction, which, in turn, disrupts the expression of genes relevant to oxidative metabolism and mitochondrial biogenesis^91,92^. The potential effects of versican reduction on mechano-signal transduction and mitochondrial function in dystrophic muscles warrant further investigation; especially, since defects in mitochondrial function and ATP synthesis have been well described in dystrophic muscles^50,93,94^.

In conclusion, our findings demonstrate the biological significance of versican as a therapeutic target in muscular dystrophy and highlight the positive, yet complex effects of versican reduction in dystrophic *mdx* mice. Follow up investigations targeting versican in dystrophic skeletal and cardiac muscles are required to build on these findings and these investigations need to employ genetic or pharmacological strategies that bypass the effects of versican reduction on embryonic development.

## Methods

### Ethics approval and mouse husbandry

This study was approved by the Animal Ethics Committees at Deakin University (A79/2011 and G06/2015). Animal care and experimental procedures were conducted in accordance with the Australian Code of Practice for the Care and Use of Animals for Scientific Purposes. Female *mdx* (C57BL/10ScSn-Dmd^*mdx*^/Arc) mice, obtained from the Animal Resource Centre (Canning Vale, WA, Australia), were bred with male hdf (heart defect) mice. The hdf mice were obtained from Hoffman-La Roche Pharmaceuticals and are haploinsufficient for the versican allele^59^. The resulting F1 *mdx* and *mdx*-hdf male pups were confirmed through genotyping and demonstrated the expected Mendelian genetic ratios. All mice were maintained in grouped cages (2–5 mice per cage) on an alternating 12 h light/dark cycle, at 21 ± 2 °C temperature, and 40–70% relative humidity. Water was provided ad libitum and mice were fed with standard mouse chow. Experimental procedures were completed on mice at 20 to 26 weeks of age.

### Echocardiography

Mice were anesthetized by inhalation of 1.5% isoflurane. Echocardiography was performed using a HD15 Purewave Ultrasound System (Phillips). All functional parameters were measured in M-mode during systole (s) and diastole (d), and included interventricular septal dimension (IVSd, IVSs), left ventricular internal diameter (LVIDd, LVIDs), left ventricular posterior wall dimensions (LVPWd, LVPWs). From these stroke volume (SV), ejection fraction (EF), and fractional shortening were calculated.

### Whole-body energy balance and metabolism and body composition

All mice underwent indirect calorimetry (Fusion Metabolic System; AccuScan Instruments). Mice were individually placed in metabolic cages and were acclimatized for 3 h prior to measurements being recorded for 24 h. Energy expenditure and substrate oxidation rates were determined using the equations by Ferrannini^95^. Spontaneous physical activity was also measured using infrared sensors within the metabolic cages (Animal Activity Meter: Opto-Varimex-Mini; Columbus Instruments). Immediately prior to muscle function testing, conscious mice were weighed and placed in a rodent MRI (Body Composition Analyzer ESF-005, EchoMR) to determine lean and fat mass.

### Ex vivo diaphragm muscle function testing

Mice were anesthetized with medetomidine (0.5 mg/kg), midazolam (5 mg/kg) and fentanyl (0.05 mg/kg), administered via an IP injection in approximately 1 ml sterile saline, until unresponsive to tactile stimuli. Blood was collected by cardiac puncture for analysis of serum creatine kinase (CK) activity. A diaphragm muscle strip (~ 5 mm wide) was excised from the linear muscle fibers in the left costal region of the diaphragm and prepared for contractile function testing, as previously described^96^. Briefly, braided surgical silk (6/0) was tied to the central tendon and rib, and then the diaphragm muscle strip was transferred to an organ bath filled with Krebs Ringer solution (137 mM NaCl, 24 mM NaHCO_3_, 11 mM D-glucose, 5 mM KCl, 2 mM CaCl_2_, 1 mM NaH_2_PO_4_H_2_O, 1 mM MgSO_4_, 0.025 mM d-tubocurarine chloride; Sigma Aldrich), bubbled with Carbogen (5% CO_2_ in O_2_; BOC Gases) and maintained at 25 °C^96^. The central tendon was tied to an immobile pin, while the rib was attached to the lever arm of a dual mode force transducer (300-CLR; Aurora Scientific). Diaphragm muscle strips were stimulated via two platinum electrodes that flanked the length of the muscle^97^. All stimulation parameters and contractile responses were controlled and measured using Dynamic Muscle Control Software (DMC v5.415), with an on-board controller interfaced with the transducer control/feedback hardware (Aurora Scientific) ^97^. Following determination of optimal length (L_o_), the maximal force producing capacity for the diaphragm muscle was determined from a force frequency curve ranging from 1 to 120 Hz, with 2 min rest in between each stimulation. Fatigability and force recovery were assessed following 4 min of rest. Specifically, the diaphragm was stimulated at 60 Hz every 5 s for 4 min and then again at 2, 5 and 10 min post fatigue testing.

Following completion of function testing, the diaphragm muscle strips were trimmed of central tendon and rib, weighed and snap frozen in liquid nitrogen. Overall muscle cross-sectional area was determined by dividing the muscle mass by the product of optimum fiber length (L_f_ which is equal to L_o_ in diaphragm muscle strips) and 1.06 mg·mm^–3^, the density of mammalian muscle. All P_o_ values were normalized for muscle cross-sectional area and expressed as specific force (sP_o_).

A 10 mm wide diaphragm strip was excised from the linear muscle fibers in the right costal region of the diaphragm and frozen in thawing isopentane for histology and immunohistochemistry. The remainder of the costal diaphragm muscle was snap frozen for biochemical analysis. Heart weight was also recorded. All samples were stored at -80 °C.

### Serum Creatine Kinase (CK) Activity

Serum CK activity was determined using a commercially available assay kit (ab155901; Abcam), as per manufacturer’s instructions.

### Histology and wheat germ agglutinin staining (WGA)

Transverse 8 μm thick frozen sections were cut from diaphragm muscle strips. Hematoxylin and eosin (H&E; Sigma-Aldrich) staining was used for muscle morphometric analysis^97^. Digital images of H&E stained muscle were captured at 200 × magnification (DM1000 upright microscope, Leica). All histology image analysis was completed using Image-Pro Plus software (Media Cybernetics). Muscle fibre size is expressed as minimal ferret diameter to control for variation in the orientation of the muscle cross-section.

WGA is an effective tissue marker for fibrosis^67^, due to the presence of WGA binding sites in the pericellular and interstitial matrices which in dystrophic muscles are enriched with collagen, proteoglycans, and glycosaminoglycans (e.g. hyaluronan)^64,66^. Diaphragm cross-sections were fixed in 4% PFA and stained with WGA conjugated with Alexa Fluor 594 (Thermo Fisher Scientific; 1:50 dilution in PBS) for 15 min. Nuclei were counter-stained with DAPI. Two non-overlapping images for each cross-section were captured on an Olympus 1X71 Inverted Fluorescence Microscope with an XM10 camera. To determine the percentage area of fibrosis in the diaphragm cross-sections, planimetric analysis of the digital images was completed using Image-Pro Plus software (Media Cybernetics)^14,98^.

### Immunohistochemistry

Immunohistochemistry for V0/V1 versican (anti-GAGβ; Millipore, AB1033) and versikine (anti-DPEAAE neo-epitope; Thermo Fisher Scientific, PA1-1748A) was performed as previously described^22,26^. For analysis of V0/V1 versican and versikine immunoreactivity, four non-overlapping representative digital images were captured with a confocal microscope of each muscle cross-section at 600 × magnification (Olympus Fluoview FV10i). To determine the percentage of muscle cross-section immunoreactive for versican or versikine, planimetric analysis of the digital images was completed using Image-Pro Plus software (Media Cybernetics)^14,98^.

To demonstrate that versican synthesis and remodelling are associated with inflammation and regeneration in dystrophic diaphragm muscles, serial sections were used to co-localize versikine with desmin or CD68. Desmin is expressed in activated satellite cells and newly regenerated muscle fibres^55^, whilst CD68 is expressed by infiltrating monocytes and macrophages in various models of muscle damage, includes dystrophic muscles from *mdx mice* and patients with DMD^56,57,99,100^. Serial sections were used, because the anti-verskine, anti-CD68 (Abcam; ab125212) and anti-desmin (Abcam; ab15200) antibodies were all raised in the same species (rabbit). Immunohistochemistry for desmin was performed as previously described^22,26^, and for CD68 as described below. A secondary Alexa Fluor 594 goat anti-rabbit antibody (Thermo Fisher Scientific; A32740; diluted in 1:1,000) was used to detect versikine and a secondary Alexa Fluor 488 goat anti-rabbit antibody (Thermo Fisher Scientific; A11034; diluted in 1:1,000) was used to detect desmin or CD68. Nuclei were counterstained with DAPI. For the co-localization experiments, representative digital images of diaphragm muscle cross-sections were captured with a confocal microscope at 600 × magnification (Olympus; Fluoview FV10i). Co-localization was confirmed on the basis of tissue morphology, hence phase images were captured and overlaid with the corresponding fluorescent images.

To support the co-localisation of versikine with inflammatory cells, and macrophages in particular, diaphragm cross-sections were co-reacted with an anti-F4/80 antibody raised in rats (Abcam, ab6640; diluted 1:100) and the anti-versikine antibody or the anti-CD68 antibody for 1 h. Followed by incubation with a secondary Alexa Fluor 594 goat anti-rabbit secondary antibody (diluted in 1:1,000) and a secondary Alexa Fluor 488 goat anti-rat secondary antibody (Thermo Fisher Scientific; A11006; diluted in 1:1,000). Nuclei were counterstained with DAPI. A negative control diaphragm cross-section stained with goat anti-rabbit and goat anti-rat secondary antibodies was also included. Representative sections were captured at 400 × magnification with a laser scanning confocal microscope (Nikon A1Rsi).

To quantify monocyte macrophage infiltration (Fig. 8), diaphragm muscles cross-sections were reacted with an anti-CD68 primary antibody (Abcam; ab125212; diluted in 1:500) for 1 h, followed by incubation with an anti-rabbit-HRP linked secondary antibody (Jackson Labs; #111035003; diluted 1:1,000)^101^. The 3-amino-9-ethylcarbazole (AEC; Sigma Aldrich; AEC101) substrate chromogen was used to visualise CD68 positive cells which stained the cytoplasm brownish-red. Nuclei were counterstained with Mayer’s haematoxylin. Three digital images were captured of each diaphragm cross-section at 200 × magnification (DM1000 upright microscope, Leica). CD68 positive cells positive cells were manually counted and expressed as number of cells per mm^2^ of muscle cross-section.

Immunohistochemistry for MyHC type I and IIa fibers was completed following the protocol described by Bloemberg and Quadrilatero^102^, using anti-MyHC I (BA-F8; DSHB; lot: 11515–43ug/ml; diluted 1:20) and anti-MyHC IIa (SC-71; DSHB; lot: 81315–65ug/ml; diluted 1:50). Following a 1 to 2 h of incubation with the MyHC primary antibodies, sections were reacted with an Alexa Fluor 350 goat anti-mouse IgG2b (Thermo Fischer Scientific; A21140; diluted 1:500) for MyHC1 and Alexa Fluor 488 goat anti-mouse IgG1 (Thermo Fischer Scientific; A21121; diluted 1:500) for MyHC IIa. To determine the number of MyHC type I or type IIa positive fibers per mm^2^ of muscle cross-section, two images per cross-section were captured at 200 × magnification using a fluorescent light imager (Zoe; Bio-Rad). All image analysis was completed using Image-Pro Plus software (Media Cybernetics).

### Collagen content

A hydroxyproline assay was used to determine the total collagen content of dystrophic diaphragm muscles^68^. Briefly, 10 mg of tissue was homogenized in 100 μl of PBS. Following the addition of 100 μl of 12 M HCl, samples were hydrolysed overnight at 105 ^○^C. To quantify collagen content, 20 μl of muscle or standards (serial dilutions of 0.1 mg/ml of hydroxyproline in 1 mM HCl) were added to a 96-well plate, dried at 60 ^○^C, and followed by the addition of 100 μl of 1.4% freshly prepared chloramine-T solution. After a 5 min incubation at room temperature, 100 μl of a 4-(dimethylamino) benzaldehyde (DMAB) was added to each well. Samples were the incubated for another 90 min at 60 ^○^C and then read at 550 nm on a spectrophotometer. Results are reported as μg of hydroxyproline per mg of wet weight tissue.

### Real time quantitative PCR (qPCR)

Diaphragm muscles were homogenized in TRIzol reagent (Thermo Fischer Scientific; 15,596,026) using a handheld homogeniser as previously described^14^. Briefly, total cellular RNA was extracted and purified using a RNeasy Mini Kit (Qiagen). An iScript cDNA synthesis kit (Bio-Rad) was used to reverse transcribe 0.25 μg of total RNA. Quantitative RT-PCR was performed using IQ SYBR Green Super mix (Bio-Rad) and oligonucleotide primers for the genes of interest (Supplementary Table 1)^98^. cDNA concentrations were determined using Quant-iT OliGreen ssDNA reagent (Thermo Fisher Scientific), and Ct values were normalized to cDNA content.

## Statistics

All data are presented as mean ± SEM with Gaussian distribution assumed. An independent sample *t*-test or a 2-way General Linear Model (GLM) ANOVA, followed by Tukey's post hoc analysis where appropriate, were performed as indicated. All statistical analyses were performed using Minitab statistical software v17 (Sydney, AUS), with p  < 0.05 being statistically significant.

## Supplementary information

Supplementary information.

## Abbreviations

½ RT: ½ Relaxation time
ADAMTS: A disintegrin-like and metalloproteinase with thrombospondin type-1 motifs
CK: Creatine kinase
CS: Chondroitin sulphate
DAPC: Dystropin-associated protein complex
ECM: Extracellular matrix
GAG: Glycosaminoglycan
Lo: Optimal length
MCP-1: Macrophage chemoattractant protein-1
RER: Respiratory exchange rate
sP_o_: Specific force
sP_t_: Specific twitch force
TGFβ: Tumor growth factor beta
TPT: Time to peak tension
