# Supplementary material for: Genetic reduction of the extracellular matrix protein versican attenuates inflammatory cell infiltration and improves contractile function in dystrophic mdx diaphragm muscles
Source: Sci Rep. 2020 Jul 6;10:11080. doi: 10.1038/s41598-020-67464-x (PMC7338466; doi:10.1038/s41598-020-67464-x)
Supplement: Supplementary file 1 — Supplementary information. [file 41598_2020_67464_MOESM1_ESM.pdf]

Title: Genetic reduction of the extracellular matrix protein versican attenuates inflammatory cell infiltration and improves contractile function in dystrophic *mdx* diaphragm muscles

Authors: Natasha L. McRae [a], Alex B. Addinsall [a, b], Kirsten F. Howlett [c], Bryony McNeill [a], Daniel R. McCulloch [a], and Nicole Stupka [a, d, *]

[a] Centre for Molecular and Medical Research, School of Medicine, Deakin University, Waurn Ponds VIC 3216, Australia

[b] Department of Physiology and Pharmacology, Karolinska Institute, Stockholm, Sweden.

[c] Institute of Physical Activity and Nutrition, School of Exercise and Nutrition Sciences, Deakin University, Geelong VIC 3216, Australia.

[d] Australian Institute for Musculoskeletal Science (AIMSS), The University of Melbourne and Western Health, St. Albans, VIC, Australia.

**** Corresponding Author:*** **Nicole Stupka, PhD**

Australian Institute for Musculoskeletal Science (AIMSS), The University of Melbourne and Western Health, St. Albans, VIC, Australia.; nicole.stupka@unimelb.edu.au.

Supplementary Tables

Supplementary Table 1: Primer sequences

Name	Forward Sequence (5`-3`)	Reverse Sequence (5`-3`)
Adamts-5	GCTACTGCACAGGGAAG	GCCAGGACACCTGCATATTT
Biglycan	GAGATTCCTCCCAACCTGCC	GGATTCCCGCCCATCTCAAT
Col1a1	TCATCGTGGCTTCTCTGGTC	GACCGTTGAGTCCGTCTTTG
Col3a1	GCCCACAGCCTTCTACAC	CCAGGGTCACCATTTCTC
Col4a1	TGTGGATCGGCTATTCCTTC	ATGGGGCGCTTCTAAACTCT
Decorin	TCATAGAACTGGGCGGCAAC	AGTAGGCAGACCTTGAGGGAT
F4/80	AAGCATCCGAGACACACACA	GGCAAGACATACCAGGGAGA
Has2	GGGACCTGGTGAGACAGAAG	ATGAGGCAGGGTCAAGCATA
Mcp-1	CCCAATGAGTAGGCTGGAGA	TCTGGACCCATTCCTTCTTG
Myogenin	TCGGTCCCAACCCAGGA	GCAGATTGTGGGCGTCTGTA
Tgfb1	GCCTGAGTGGCTGTCTTTTGA	CACAAGAGCAGTGAGCGCTGAA
V0/V1 Vcan [1]	ACCAAGGAGAAGTTCGAGCA	CTTCCCAGGTAGCCAAATCA

[1] The versican primers specifically detect the V0 and V1 isoforms.

Supplementary Figures

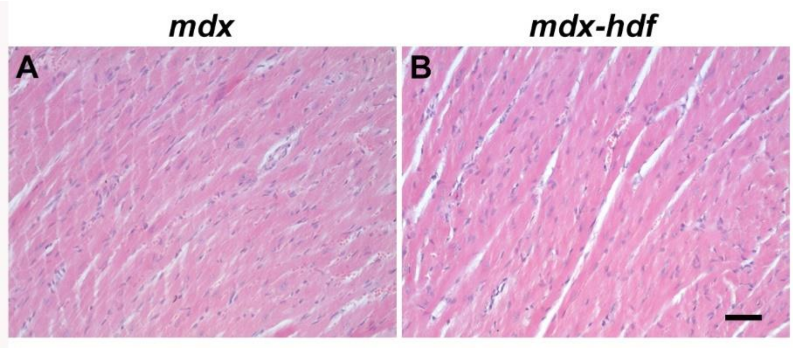

Fig. S1: H&E stained myocardium from *mdx* and *mdx*-hdf mice. There was no histological evidence of fibrosis in hearts from either *mdx* or *mdx*-hdf mice. Scale bar = 100 µm.
